# Lyophilized Composite Loaded with Meloxicam-Peppermint oil Nanoemulsion for Periodontal Pain

**DOI:** 10.3390/polym13142317

**Published:** 2021-07-14

**Authors:** Amal M. Sindi, Khaled M. Hosny, Waleed S. Alharbi

**Affiliations:** 1 Department of Oral Diagnostic Sciences, Faculty of Dentistry, King Abdulaziz University, Jeddah 21589, Saudi Arabia; amsindi@kau.edu.sa; 2 Department of Pharmaceutics, Faculty of Pharmacy, King Abdulaziz University, Jeddah 21589, Saudi Arabia; wsmalharbi@kau.edu.sa

**Keywords:** meloxicam, self-nano emulsifying, lyophilized tablet

## Abstract

Maintaining oral health helps to prevent periodontal inflammation and pain, which can progress into more detrimental issues if left untreated. Meloxicam (MX) is a commonly used analgesic for periodontal pain, but it can have adverse gastrointestinal effects and poor solubility. Therefore, this study aimed to enhance the solubility of MX by developing a self-nanoemulsifying drug delivery system (SNEDDS). Considering the anti-ulcer activity of peppermint oil (PO), it was added in a mixture with medium-chain triglyceride (MCT) to the MX-loaded SNEDDS formulation (MX-PO-SNEDDS). After optimization, MX-PO-SNEDDS exhibited a PO:MCT ratio of 1.78:1, surfactant mixture HLB value of 14, and MX:oil mix ratio of 1:15, a particle size of 47 ± 3 nm, stability index of 85 ± 4%, ex vivo J_ss_ of 4 ± 0.6 μg/cm^2^min, and ulcer index of 1 ± 0.25 %. Then, orally flash disintegrating lyophilized composites (MX-SNELCs) were prepared using the optimized MX-PO-SNEDDs. Results reveal that MX-SNELCs had a wetting time of 4 ± 1 s and disintegration time of 3 ± 1 s with a high in vitro MX release of 91% by the end of 60 min. The results of pharmacokinetic studies in human volunteers further demonstrated that, compared to a marketed MX tablets, MX-SNELCs provided a higher C_max_, T_max_, and AUC and a relatively greater bioavailability of 152.97 %. The successfully developed MX-SNELCs were found to be a better alternative than the conventional tablet dosage form, thus indicating their potential for further development in a clinically acceptable strategy for managing periodontal pain.

## 1. Introduction

Oral health is an important constituent of overall well-being that includes social, economic, and psychological aspects [1]. A broader meaning of oral health implies a condition without any disease or resisting any disease, with socially acceptable appearance, capable of mastication of food, and permitting the individual in a desired social role [2]. Moreover, maintaining oral health can prevent problems, such as gingivitis, and subsequent periodontal disease and pain [3]. Initially, gingivitis manifests as inflammation of periodontal tissues and may be without any pain; however, in later stages it can cause tissue damage and even tooth loss [4].

Meloxicam (MX) is an analgesic and anti-inflammatory agent that belongs to the class of non-steroidal anti-inflammatory drugs (NSAIDs) [5]. Specifically, it is a selective cyclooxygenase-2 (COX-2) enzyme inhibitor with a therapeutic effect comparable to other NSAIDs but with lesser gastrointestinal adverse effects [5]. In a reported comparative study, a topical MX gel exhibited lesser systemic side effects and better performance than diclofenac and piroxicam gels against pain and inflammation [6]. Nevertheless, inducing gastrointestinal ulcers remains a challenge for the oral delivery of MX, and poor aqueous solubility also limits the development of novel MX formulations [7]. Therefore, this work proposes a self-nanoemulsifying drug delivery system (SNEDDS) to enhance the solubility of MX [8].

One of the most important aspects in the development of SNEDDS is selecting the appropriate oil phase, particularly one that can complement the drug action or reduce its adverse effect. [9] Thus, using an oil phase that can decrease gastrointestinal ulceration caused by Meloxicam is desirable. Essential oils, especially peppermint oil (PO) from *Mentha × piperita*, are known to have anti-ulcer, analgesic, and anti-inflammatory activities [10,11,12]. In addition, PO is primarily composed of menthone, menthofuran, limonene, cineole, and 40% menthol, which contributes good anti-ulcer activities [9,13].

When combined in a SNEDDS, the presence of PO can promote the therapeutic efficiency and minimize the gastrointestinal ulceration effects of MX, while SNEDDS can enhance the solubility of MX. Nevertheless, PO is a volatile oil with low viscosity, which could pose problems in the formulation of SNEDDS. Therefore, the inclusion of a medium-chain triglyceride (MCT) containing oil phase in the formulation would be necessary to further solubilize MX to a higher extent than by using PO alone. After optimizing the SNEDDS, the formulation can be further converted into unit dosage forms such as composites [14].

While SNEDDSs offer the advantage of enhanced drug solubility, rapid onset of action is desirable in the case of periodontal and tooth pain, which can be achieved by converting the formula into lyophilized rapid disintegrating composites [15,16]. As a result, delivering MX in a lyophilized orodispersible composite with rapid and flash disintegration for fast onset of action in alleviating periodontal pain would be desirable. This would be particularly beneficial considering the sudden onset and acute clinical manifestations of periodontal and tooth pain [17].

To select the appropriate formulation components and strategies, several aspects of statistical experimental design need to be considered to yield the optimal product. However, a conventional one-factor-at-a-time approach is unacceptable in such a case. As an alternative, the design of experiments (DoE) is a useful tool to optimize the formulation component at any stage, to obtain the desired product responses, and is applicable for both research and industrial perspectives [18]. Therefore, we applied DoE to develop the formulation and optimize dosage forms and designed I-optimal quadratic response surface experiments for optimization.

Hence, the present study aimed to enhance the anti-inflammatory action of MX by loading it in a nanoemulsion to enhance its solubility, in vitro release, and ex vivo permeation. It was hypothesized that the formulation of SNEDDS with peppermint oil (PO) as an oil phase constituent would reduce the ulcerative side effect associated with MX, whereby menthol as the main constituent of PO has strong anti-ulcer activity. I-optimal quadratic response surface experimental design was considered for the optimization of SNEDDS formulation. It was also presumed that the preparation of orally disintegrating lyophilized composites with the optimized MX-PO SNEDDs will provide a rapid onset of action to relieve periodontal pain. 

## 2. Materials and Methods

### 2.1. Materials

Meloxicam (MX) was kindly gifted by Amoun Pharmaceutical Co. (Cairo, Egypt). Peppermint oil, propylene glycol, fumed silica, hydroxypropyl cellulose, Kollidon, spray-dried lactose, sorbitol, and gelatin were purchased from Tedia Company (Fairfield, OH, USA). Oleic acid, soybean oil, Sefsol, palm kernel oil, coconut oil, and Labrafac Lipophile WL 1349, Tween 80, Span 80, Labrasol, Triton, Cremophor, Kollifor, polyglyceryl oleate, and Plurol were generously gifted by Gattefosse (Saint-Priest, France). Polyethylene glycol (PEG) 200, PEG 400, propylene glycol, Transcutol, glycerol, and ethanol were purchased from Sigma-Aldrich (St. Louis, MO, USA). All other reagents and chemicals used were of standard analytical grades.

### 2.2. Determination of Meloxicam (MX) Solubility in Formulation Components 

The solubility of MX was estimated in various oils, surfactants, and co-surfactants. Peppermint oil, oleic acid, soybean oil, Sefsol, palm kernel oil, coconut oil, and Labrafac Lipophile WL 1349 were studied as the oil phase. Meanwhile, Tween 80, Span 80, Labrasol, Triton, Cremophor, Kollifor, polyglyceryl oleate, and Plurol were analyzed as the surfactants. The co-surfactants included polyethylene glycol (PEG) 200, PEG 400, propylene glycol, Transcutol, glycerol, and ethanol. The solubility of MX was determined by shaking an excess of MX with 2 mL of each oil, surfactant, and co-surfactant samples at 25 ± 0.5 °C for 48 h. The samples were then centrifuged at 1200 rpm for 15 min, diluted with methanol, and quantified by UV-Vis spectrophotometry. 

### 2.3. Pseudoternary-Phase Diagram in Formulation Components

A pseudoternary-phase diagram was obtained to identify the nanoemulsion region in the selected formulation systems on the basis of MX solubility. Three formulation phases were selected. The first phase was the oil mixture with PO and Labrafac Lipophile WL 1349 (represented as MCT) in the PO:MCT ratio of 1:1. The second phase included a mixture of selected surfactants, Tween 80 and Span 80 with an HLB value of 12. PEG 400 was selected as the co-surfactant for the third phase to construct the pseudoternary-phase diagram. Each combination of the three components contributed to 100% and contained 7.5 mg of MX. Finally, the nanoemulsion regions were identified in the pseudoternary-phase diagram.

### 2.4. Preparation and Optimization of MX-PO-SNEDDS 

#### I-Optimal Quadratic Response Surface Experimental Design 

After preparing MX-loaded SNEDDS using PO (MX-PO-SNEDDS), as the oil component, it was important to study the effects of various formulation components for optimization. The I-optimal quadratic response surface experimental design was employed to optimize the MX-PO-SNEDDS formulation, the details of which are provided in Table 1. Three independent factors were selected, including the PO:MCT ratio, HLB for surfactant mixture, and MX:oil mix ratio. The measured responses were particle size of emulsion droplets, stability index, ex vivo steady-state flux across the membrane (J_ss_), and the ulcer index. In total, 18 randomly ordered runs with 7.5 mg MX in each formulation were performed. About 1 g of each suggested formulation was prepared. The total of three components (oil mixture, surfactant, and co-surfactant) in the mixture was maintained equal to 100%.

### 2.5. Evaluation of MX-PO-SNEDDS Formulations

#### Determination of Particle Size of MX-PO-SNEDDS

The aqueous dispersions of the prepared formulations were diluted appropriately with distilled water, and the diluted samples were analyzed for particle size (Zetatrac, Microtrac, Montgomeryville, PA, USA).

### 2.6. Stability Index

To determine the stability index of the MX-PO-SNEDDS formulations, The samples were subjected to three consecutive freeze–thaw cycles, with a freeze temperature of −25 °C and thaw temperature of +25 °C, for 12 h. The stability index was calculated using the initial and final particle size as follows:

(1) $$\mathrm{Stability}\;\mathrm{index}\;=\frac{\mathrm{Initial}\;\mathrm{particle}\;\mathrm{size}\;–\mathrm{Change}\;\mathrm{in}\;\mathrm{size}}{\mathrm{Initial}\;\mathrm{size}}\times 100$$

### 2.7. Ex Vivo Permeation Steady-State Flux across the Membrane (J_ss_)

Ex vivo permeability studies for MX for the different MX-PO-SNEDDS formulations were performed on sheep buccal mucosa. The prepared mucosa (2 cm × 2 cm) was mounted on an automatic Franz diffusion cell (MicroettePlus, Hanson Research, Los Angeles, CA, USA) with an area of 1.76 cm^2^ and receptor cell volume of 7 mL. A known quantity of each formulation (equivalent to 7.5 mg MX) was loaded in the donor part. Phosphate buffered saline, at pH 6.8 and temperature of 32 ± 2 °C under stirring at 400–420 rpm, was employed as the receptor medium. The MX content in the samples withdrawn at predetermined time points was estimated using a high-performance liquid chromatography. The amount of MX permeation per unit area across the sheep buccal mucosa was determined and used to calculate the steady-state flux (J_ss_). 

### 2.8. Determination of Ulcer Index

The studies were conducted in male albino rats with body weights in the range of 160–240 g and approved by the Institutional Review Board for Animal Research/Studies Animals (Approval No.11-03-2021). The study animals were obtained from the animal house of the Beni Suef Center for Clinical Laboratory in Beni Suef, Egypt. Three animals were assigned for each MX-PO-SNEDDS formulation for the determination of the ulcer index. A 15-day treatment was performed on each animal with the MX-PO-SNEDDS formulation. On day 16, macroscopic examination of the excised stomach was conducted for observing hemorrhagic lesions [19]. The number of ulcers in a unit area was also determined to assess the severity microscopically. The following scores were assigned to determine the ulcer index: a score of 0.5 for a normal-colored stomach lining; 1 for red coloration of the lining; 2 for spot ulcerations; 3 for hemorrhagic streaks; 4 for ulcers between 3–5 mm in diameter; 5 for ulcers greater than 5 mm; and 6 for ulcers with perforations. For comparison, the ulcer index was also determined for the optimized formula.

### 2.9. Optimization of MX-PO-SNEDDS Formulation

The optimization of the formulation was carried out according to the constraints and goals presented in Table 2. The optimum formula was expected to provide maximum values for stability index and ex vivo J_ss_, and minimum values for particle size and ulcer index.

### 2.10. Preparation and Evaluation of Meloxicam Self-Nanoemulsion Lyophilized Composite (MX-SNELCs)

The formulation and preparation of MX-SNELCs were performed according to a previously reported method [15]. Each prepared MX-SNELC contained 7.5 mg MX.

### 2.11. Preparation of MX-SNELCs

The MX-SNELCs were achieved by mixing 1 g of the optimized MX-PO-SNEDDS, fumed silica (400 mg), hydroxypropyl cellulose (100 mg), Kollidon (400 mg), 100 mg of spray-dried lactose, and 100 mg of sorbitol, and 9 mL of 1.5% gelatin solution on a stirrer. At first, the mixing was done for 2 min by vortexing and later using a probe sonicator to obtain a homogenous blend. The prepared blend was filled into composite blister pockets and kept for 24 h at −22 °C. Then, freeze-drying was done for 24 h, at −45 °C. The final lyophilized composites were subjected to the following evaluations according to reported procedures [20].

### 2.12. Wetting Time

The wetting time of MX-SNELCs was obtained in triplicate by placing the composite onto a tissue paper (12 cm × 10.75 cm, with two folding) wetted with Sorenson’s buffer solution (pH 6.8, 6 mL)

### 2.13. Disintegration Time

The disintegration time of the six MX-SNELCs at 37 ± 0.5 °C was obtained with a disintegration tester using 250 mL of Sorenson’s buffer at pH 6.8.

### 2.14. In Vitro MX Release Study

The release of MX from MX-SNELCs was assessed in triplicate by employing a paddle-type USP dissolution test apparatus with simulated saliva fluid (500 mL) at pH 6.8 and 37 ± 0.5 °C. The test was carried out at 50 rpm, and 5 mL samples were withdrawn, filtered through a 0.45-μm filter, then quantified for MX. The medium was maintained at a fixed volume by introducing the same volume of fresh medium to compensate for the loss of volume during sampling at each time point. 

### 2.15. In Vivo Pharmacokinetic Studies

The randomized and open-labeled study, in accordance with the Helsinki agreement protocol, was approved by the Ethics Committee of the Beni-Suef Clinical Laboratory Center for clinical studies (Approval No: 07-003-2021), Egypt. The protocol followed a single dose and parallel design with a 1-day study and 14-day screening. A single MX-SNELC corresponding to 7.5 mg MX was provided to the subjects with the direction to keep the MX-SNELC in the oral cavity for 3 min before ingestion. The marketed MX composite (7.5 mg) was provided for oral administration with 250 mL water. Volunteers were directed to remain at the site for 24 h after sample administration for blood sample collection [21]. The data were processed using a non-compartmental model (PK-SOLVER^®^ software), and the desired pharmacokinetic parameters were determined.

## 3. Results and Discussion

### 3.1. Determination of Meloxicam (MX) Solubility in Formulation Components 

The solubility of MX in various oils, surfactants, and co-surfactants was determined and is shown in Table 3. Although the nanoemulsions prepared using essential oils have low viscosity, the solubility of MX in PO was found to be 440 ± 15 mg/mL. Therefore, the combined use of PO with another oil containing medium-chain triglyceride (MCT) was planned to resolve this issue. Among the oils, the highest drug solubility (715 ± 37 mg/mL) was observed in Labrafac Lipophile WL 1349; thus, it was chosen to combine with PO as the oil component for nanoemulsions. In addition, this mixture with different ratios of PO:MCT was chosen as one of the independent variables in the experimental design.

Among the surfactants studied, Span 80 provided the highest solubility of 204 ± 18 mg/mL. Because the hydrophilic-lipophilic balance (HLB) value was considered as one of the independent variables in the experimental design, it was necessary to select one more surfactant to mix with Span 80 to obtain the desired HLB value. Tween 80 (145 ± 11 mg/mL) showed the second-highest value of drug solubility and, thus, was chosen for the surfactant mixture with Span 80. This combination of Span 80 and Tween 80 was previously reported to be useful [22]. Therefore, Tween 80 was mixed with Span 80 in different proportions to obtain the described HLB value for formulation trials.

In the case of co-surfactants, the highest solubility was found in PEG 400 with 170 ± 15 mg/mL. Further, the significant enhancement of solubility of MX by PEG 400 is well established [23]. Therefore, PEG 400 was selected as the co-surfactant for the formulation of SNEDDS.

### 3.2. Pseudoternary-Phase Diagram in Formulation Components

**Figure 1:**
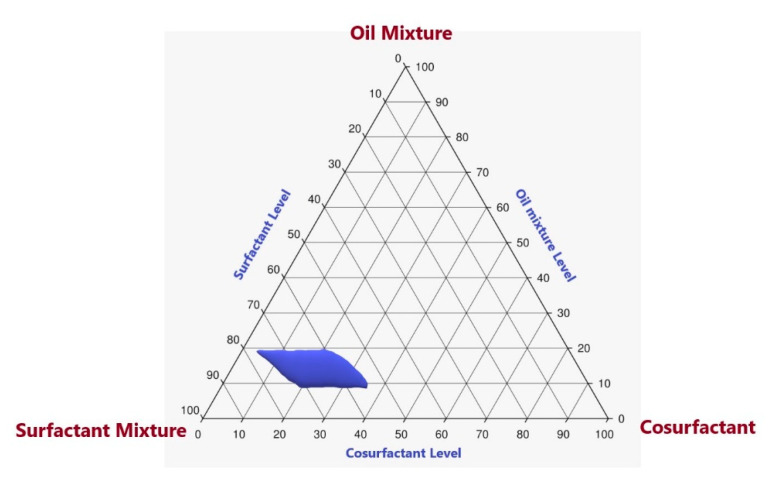
Pseudoternary-phase diagram in formulation components. The oil mixture contained peppermint oil (PO) and Labrafac Lipophile WL 1349 (MCT) in the PO:MCT ratio of 1:1. The surfactant mixture contained Tween 80 and Span 80 with an HLB value of 12. PEG 400 was the co-surfactant.

The purpose of this study was to identify the suitable levels for formulating a self-nanoemulsion component. The pseudoternary-phase diagram in Figure 1 shows that the nanoemulsion region was present when the oil mixture ranged 10–20%, surfactant ranged 55–75%, and co-surfactant ranged 5–35%. 

### 3.3. Preparation and Optimization of MX-PO-SNEDDS 

The results of the design of experiments are provided in Table 4. The particle size, stability index, ex vivo steady-state flux across membrane, and ulcer index for each run were determined by the Design Expert software. 

### 3.4. Particle Size

A quadratic response surface model was suggested for particle size by the Design Expert software on the basis of the trials. The quadratic response model had a *p*-value of < 0.0001. Further, the lack of fit was not significant (*p*-value > 0.05). Thus, the quadratic response surface model was acceptable for the particle size response. The predicted and adjusted R-squared values of 0.9782 and 0.9959 were comparable. Moreover, the fitted values for the particle size were found to have a good correlation with the observed values (Table 4).

The analysis of variance (ANOVA) data for the particle size of MX-PO-SNEDDS formulations prepared in various experimental runs are shown in Table 5. From the results, it can be seen that B, C, AC, BC, A^2^, B^2^, and C^2^ are significant model terms with a significant influence on the particle size of NE droplets.

The polynomial equation in terms of coded factors suggested by the software for particle size of MX-PO-SNEDDS formulations is provided in Equation (2). Surprisingly, the ANOVA data suggest that PO:MCT ratio has no significant effect on the particle size of the MX-PO-SNEDDS formulation. While it is generally seen that higher oil content favors larger particle size [24], the effect of the PO:MCT ratio was selected as an independent factor rather than oil concentration in the present study. Therefore, the type of oil does not have a significant influence on particle size, and it may be assumed that PO and MCT have a similar effect on particle size. Meanwhile, the HLB value and MX:oil ratio both influenced particle size but with inverse relationships, as indicated by the negative sign for the coefficients. In addition, the Equation (2) suggests a higher influence of the HLB value than the MX:oil ratio. As previously demonstrated, particle size was reduced at higher HLB values when prepared by an inverse microemulsion method [22]. Interestingly, since the presented study also used a combination of Tween 80 and Span 80, it could be assumed that a similar effect was obtained in the MX-PO-SNEDDS formulations. Nevertheless, an HLB value matching the requirement of the oil phase is considered most appropriate for stable micro and nanoemulsion systems [25]. For the MX:oil mix ratio, a higher value denotes lower oil content and vice versa. The observed results indicate that a higher MX:oil mix ratio favors lower particle size, or in other words, lower oil content reduces particle size, which agrees with the literature [24]. 

**Figure 2:**
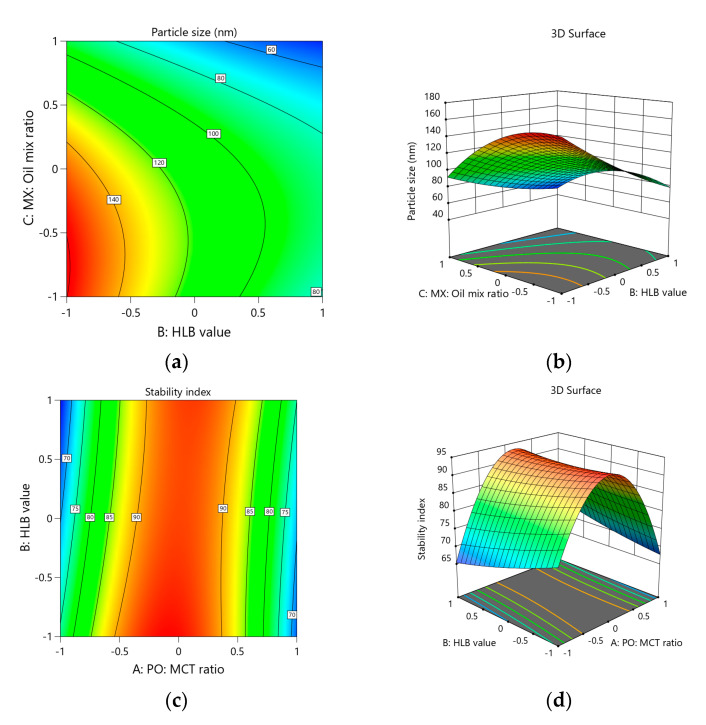

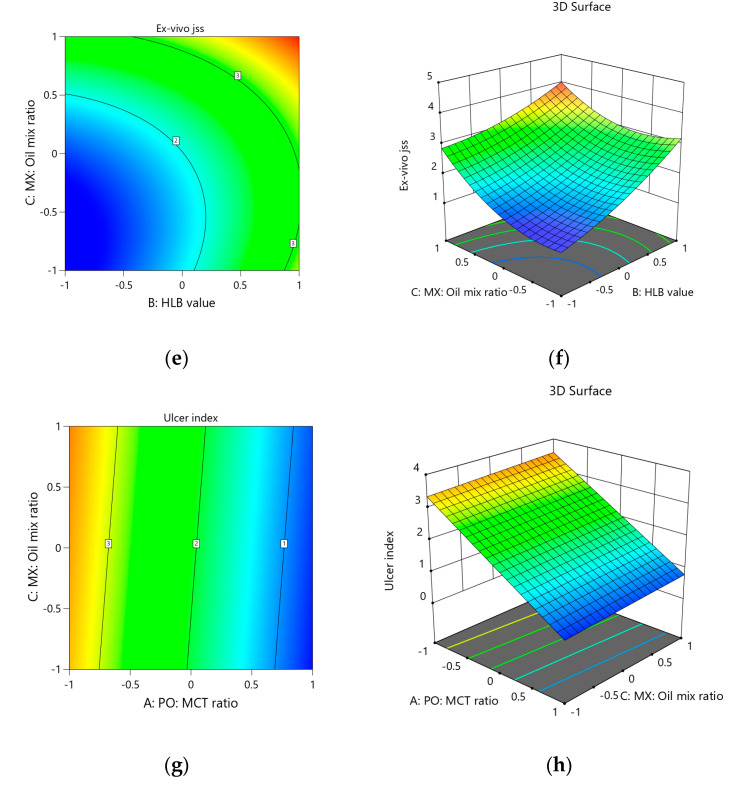
Design plots of the various responses: (**a**) contour plot for particle size, (**b**) response surface plot for particle size, (**c**) contour plot for stability index, (**d**) response surface plot for stability index, (**e**) contour plot for ex vivo J_ss_, (**f**) response surface plot for ex vivo J_ss_, (**g**) contour plot for ulcer index, and (**h**) response surface plot for ulcer index.

In addition to the above-mentioned individual effects, it was found that the terms AC, BC, A^2^, B^2^, and C^2^ also exhibited a significant influence on particle size. Thus, it is pertinent to consider the overall effect of the individual and interactions effects on particle size. The results for which are presented as contour and response surface plots of the various responses in Figure 2a,b. These plots reveal that the HLB value effect on particle size is influenced by the MX:oil mix ratio and vice versa. At lower values of the MX:oil ratio, the influence of HLB value on particle size was almost linear and was slightly less at higher MX:oil mix ratios. A similar effect was observed for MX: Oil mix ratio on changing the HLB value.

Particle size = 111.39 + 1.44 × A − 31.20 × B − 24.46 × C + 0.5257 × AB − 3.10 × AC + 9.70 × BC − 4.23 × A^2^ + 5.57 × B^2^ − 22.17 × C^2^ (2)

### 3.5. Stability Index

To evaluate the stability index, a quadratic model was established by the software with a significant *p*-value < 0.0001 and the lack of fit was not significant (*p*-value = 0.1139). Overall, the design model was acceptable for the optimization of the formulation. The predicted and adjusted R-squared values were determined to be 0.9572 and 0.9851, respectively, and the fitted values for the stability index showed good correlation with the observed values (Table 4).

The ANOVA data for the stability index are presented in Table 6. From the results, it can be seen that C, AB, AC, and A^2^ are significant model terms with a significant influence on the stability index. 

The polynomial equation in terms of coded factors suggested by the software for stability index is provided in Equation (3). The equation implies that the PO:MCT ratio and HLB value had no significant influence on the stability index. From this result, it can be derived that all the MX-PO-SNEDDS formulations suggested in the design run were stable at the selected levels of PO:MCT ratio and HLB value and were not affected by the freeze–thaw cycles. Nevertheless, the MX:oil mix ratio (term C) had an inverse effect on the stability index, as indicated by a negative coefficient value. A higher stability index value indicates higher stability of the formulation in terms of change in globule size. Thus, based on the individual effects, increasing the MX:oil mix ratio decreases the stability index. The ANOVA data also indicate that the terms AB, AC, and A^2^ are also significant, and thus, the overall effect on the stability index depends on the interactions of the independent factors. These results are presented as contour and response surface plots in Figure 2c,d, respectively, which show that the stability index first increased then later decreased as the MX:oil mix ratio increased. At around the midpoint of the selected level of MX:oil mix ratio, a maximum value for stability index was observed. This means that an optimum value for the MX:oil mix ratio is necessary to achieve a formulation with high stability. Thus, the individual effect of the MX:oil mix ratio was significantly influenced by the terms AB, AC, and A^2^.

Stability index = +92.99 + 0.4197 × A − 0.7622 × B − 1.09 × C + 4.42 × AB + 2.95 × AC + 0.9879 × BC − 22.75 × A^2^ + 0.7508 × B^2^ − 1.42 × C^2^ (3)

### 3.6. Ex Vivo Permeation Steady-State Flux Across the Membrane (J_ss_)

A quadratic model suggested for ex vivo J_ss_ exhibited a *p*-value of < 0.0001 and a lack of fit *p*-value of 0.1131. Thus, the model was acceptable for the evaluation and optimization of the MX-PO-SNEDDS formulation in terms of ex vivo J_ss_. The predicted and adjusted R-squared values were 0.9363 and 0.9867, respectively. Further, both the observed values and predicted values were close to each other (Table 4). All parameters indicate that the design model is suitable for the optimization of the formulation. The ANOVA data for ex vivo J_ss_ in Table 7 show that B, C, AC, BC, A^2^, B^2^, and C^2^ are significant model terms with a significant influence on ex vivo J_ss_. 

Equation (4) presents the polynomial equation suggested by the software for ex vivo J_ss_ in terms of coded factors. The factor PO:MCT ratio, once again, proved to not significantly influence a response, which means that ex vivo J_ss_ is not significantly affected by the PO:MCT ratio. In other words, both PO and MCT have a similar influence on ex vivo J_ss_. This result was unexpected as PO and its terpene constituents are well-established permeation enhancers [26,27]. Although, it has also been reported that MCT containing Labrafac lipophile WL 1349 is a good permeation enhancer [28]. Therefore, it is possible that the PO and Labrafac lipophile WL 1349 had similar permeation enhancement effects resulting in similar ex vivo J_ss_.

In addition, the HLB value and MX:oil mix ratio had a significant influence on ex vivo J_ss_, which both increased the value of the response. Comparatively, the HLB value had slightly greater influence on ex vivo J_ss_ than the MX:oil mix ratio. It has been reported that mixed surfactants provide better permeation effects for o/w nanoemulsions [29]. Thus, the combined use of Tween 80 and Span 80 might have contributed to the enhancement of ex vivo J_ss_. Moreover, a higher MX:oil mix ratio indicates a greater drug concentration in the donor compartment compared to the oil phase. This might be the reason for the higher flux at a higher MX:oil mix ratio. While the interaction terms AC, BC, A^2^, B^2^, and C^2^ are also significant, the contour and response surface plots (Figure 2e,f) for the response show that there are no major deviations from the individual effects of the HLB value and MX:oil mix ratio. In these plots, it can be seen that lower concentrations of both HLB value and MX: Oil mix ratio favor lower values of ex vivo J_ss_ and vice versa.

Ex vivo J_ss_ = 1.99 − 0.0143 × A + 0.8271 × B + 0.6592 × C − 0.0739 × AB + 0.1123 × AC − 0.2454 × BC + 0.1469 × A^2^ + 0.2302 × B^2^ + 0.5592 × C^2^ (4)

### 3.7. Determination of Ulcer Index

Unlike particle size, stability index, and ex vivo J_ss_, a linear model was suggested by the software for the ulcer index (*p*-value < 0.0001). The predicted and adjusted R-squared values were 0.9218 and 0.9441, respectively. Further, the fitted values for the ulcer index were found to have a good correlation with the observed values (Table 4). The ANOVA data for the ulcer index are shown in Table 8. 

Equation (5) can be used to calculate the ulcer index in terms of coded factors, as suggested by the software. From the ANOVA data, it can be seen that only the PO:MCT ratio (term A) had a significant effect (*p*-value < 0.0001) on the ulcer index. As previously mentioned, PO and its constituents, such as menthol, limonene, and cineole, have significant anti-ulcer activities [9]. Thus, as the proportion of PO increases in the oil phase, a lower ulcer index can be expected. This was confirmed by the negative sign of the coefficient of term A, and thus, a higher PO:MCT ratio results in a lower ulcer index. This effect is clearly observed in the contour and response surface plots (Figure 2g,h), where the iso-value curves in the contour plot are almost perpendicular to the PO:MCT ratio. Meanwhile, the response curve shows a dip towards higher values of the PO: MCT ratio. Both of these observations confirm that the ulcer index decreased as the PO:MCT ratio increased.

Ulcer index = 2.06 − 1.38 × A − 0.0448 × B + 0.1063 × C (5)

### 3.8. Optimization of MX-PO-SNEDDS Formulation

As shown in Table 9, the optimum formula suggested by the software is comprised of 75 mg MX, 112.5 mg oil mixture (72 mg PO + 40.5 mg Labrafac Lipophile), 650 mg surfactant mix with an HLB value of 14 (58.5 mg Span 80 + 591.5 mg Tween 80), and 230 mg PEG 400. The predicted and observed values of the responses are also shown in Table 9.

### 3.9. Preparation and Evaluation of Meloxicam Self-Nanoemulsion Lyophilized Composite (MX-SNELCs)

#### Preparation of MX-SNELCs

**Figure 3:**
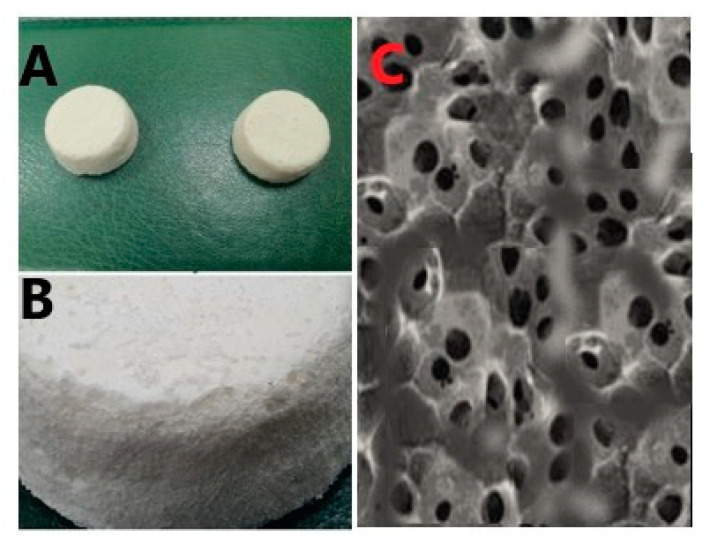
Photographs (**A**,**B**), and SEM image (**C**) for the prepared MX-SNELCs.

The photographs and SEM images of the prepared MX-SNELCs in Figure 3 reveal the porous nature of the lyophilized composites. The gelatin, surfactants, sugars, and polymers can deeply influence the porosity of the prepared composites, whereby the microstructure of lyophilized composites with surfactants can have deep pores and channels [30]. Further, polyhydric alcohols or sugar alcohols can enhance pore size in lyophilized composites prepared with gelatin [31]. Thus, the porous surface morphology of the prepared MX-SNELCs is reasonable.

### 3.10. Wetting Time

A very low wetting time of 4 ± 1 s was observed for the MX-SNELCs. The presence of surfactants and co-surfactant would have contributed significantly to this high wettability of the system. Interestingly, this value was significantly lower than the reported value of 50–180 s for lyophilized composites of a similar system [20]. The presence of Kollidon, spray-dried lactose, and sorbitol could be the reason for a lower wetting time. All these are useful as wetting agents in pharmaceutical formulations.

### 3.11. Disintegration Time

A disintegration time of 3 ± 1 s was observed for the MX-SNELCs. A faster wetting of the composite could result in rapid swelling of the disintegrating agent and, thus, decrease disintegration time, which was seen for the MX-SNELCs. A similar system demonstrated a disintegration time in the range of 3 – 60 s. Further, a high influence of emulsifiers on the disintegration time of lyophilized composites is already established [20]. In another study, Tween 80/Span80 blend reduced the disintegration of lyophilized composites to only a few seconds [30]. As surfactants reduce the interfacial tension and increase the wettability, using a surfactant mixture containing Tween 80 and Span 80 in the present study might have also contributed to the very fast disintegration MX-SNELCs. Previously, sorbitol was found to increase the hardness of lyophilized composites at lower concentrations but decreased the composite hardness and disintegration time at higher concentrations [31]. Thus, it could be assumed that the concentration of sorbitol was sufficiently high enough to reduce the disintegration time of MX-SNELCs in the present study.

### 3.12. In Vitro MX Release Study

**Figure 4:**
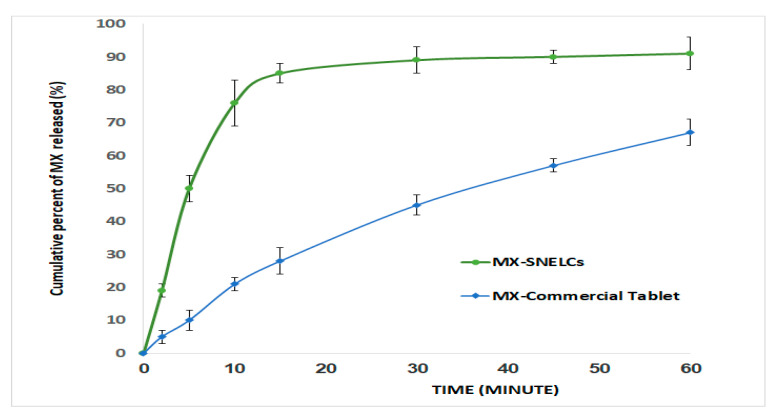
In vitro drug release profiles of lyophilized composite loaded with Meloxicam SNEDDS prepared with peppermint oil (MX-SNELCs) and marketed composite of Meloxicam (MX commercial tablets).

The in vitro MX release profile for the MX-SNELCs in Figure 4 revealed the MX dissolution from MX-SNELCs was significantly greater than that from the marketed product. An initial burst release of MX was observed from MX-SNELCs, then around 50% of the drug was released after 5 min and 91% was released after 60 min. These are notable results compared to the marketed product that demonstrated slower release and lower drug dissolution at all intervals with no significant burst release. Similar observations were reported for lyophilized composites of a griseofulvin dry emulsion [30]. Since higher wetting and disintegration can improve drug dissolution, an enhancement in drug dissolution could be expected for the prepare MX-SNELCs. Thus, the presence of the surfactant mixture containing Tween 80 and Span 80 might have a significant effect on the enhanced dissolution of MX from MX-SNELCs due to the significant reduction of interfacial tension [30,32].

### 3.13. In Vivo Pharmacokinetic Studies

**Figure 5:**
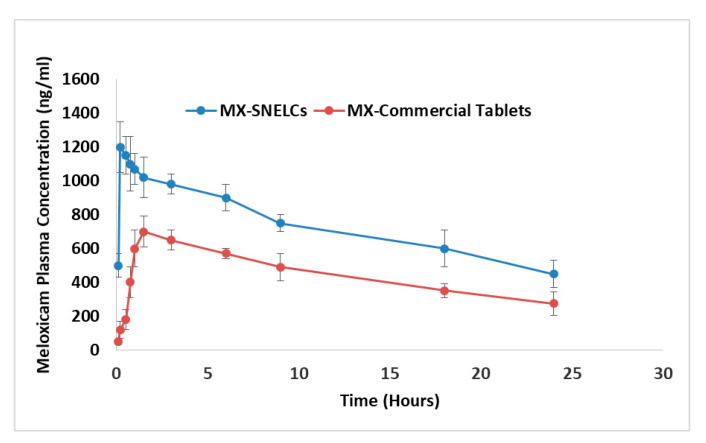
In vivo pharmacokinetics of lyophilized composite loaded with Meloxicam SNEDDS prepared with peppermint oil (MX-SNELCs) and marketed tablet of Meloxicam (MX commercial tablets).

The pharmacokinetic profiles of MX in human volunteers after administration of the prepared MX-SNELCs and marketed tablet of MX are shown in Figure 5. A higher MX bioavailability from MX-SNELCs could be easily identified from the plasma drug concentration–time profiles. The plasma MX concentration was higher from MX-SNELCs at all time points compared to that from the marketed tablet. Results of in vivo pharmacokinetic data (Table 10) indicate that MX-SNELCs enhanced the bioavailability of MX by 1.52 folds, shortened the onset of action to less than 5 min, increased the maximum plasma level, and reduced the T_max_ compared to the marketed MX tablet formulation. A similar enhancement in the bioavailability of lyophilized composites was reported in previous studies [20,33]. Moreover, the fast disintegration and high drug release may have also contributed to the enhanced bioavailability of MX-SNELCs compared to marketed tablets.

## 4. Conclusions

The present study reports the preparation of a lyophilized tablet loaded with MX-PO-SNEDDS (MX-SNELCs) with enhanced bioavailability and effectiveness for clinical use. The optimized formulation of MX-PO-SNEDDS contained an PO:MCT ratio of 1.78:1, surfactant mixture HLB value of 14, and MX:oil mix ratio of 1:15 in addition to a particle size, stability index, ex vivo J_ss_, and ulcer index of 47 ± 3 nm, 85 ± 4 %, 4 ± 0.6 μg/cm^2^⋅min, and 1 ± 0.25%, respectively. Subsequently, MX-SNELCs were prepared using the optimized MX-PO-SNEDDs (1 g), fumed silica (400 mg), hydroxypropyl cellulose (100 mg), Kollidon (400 mg), spray-dried lactose (100 mg), sorbitol (100 mg), and a gelatin solution (9 mL of 1.5%). Results reveal a fast wetting and rapid disintegration of MX-SNELCs, which released around 91% of MX after 60 min according to the in vitro release study. In addition, MX-SNELCs demonstrated a significant improvement in pharmacokinetic parameters of MX compared to a marketed MX tablet, specifically enhancing the bioavailability of MX by 1.52 folds. Overall, the low ulcer index of MX-PO-SNEDDs and enhanced bioavailability of MX-SNELCs suggest that our proposed formulation is promising for future use in the clinical management of periodontal pain. Of course, it will not obviate the need for further clinical evaluation regarding the bioactivity and toxicity for this novel formula, which may provide clinicians with other important data.

## Figures and Tables

**Table 1:** The independent factors and responses for the design of experiments.

Run	Coded Values			Actual Values		
	Factor 1	Factor 2	Factor 3	Factor 1	Factor 2	Factor 3
	A:PO: MCT Ratio	B:HLB Value	C:MX: Oil mix Ratio	A:PO: MCT Ratio	B:HLB Value	C:MX: Oil Mix Ratio
1	1	−1	−1	2:1	10	1:25
2	0.14	0.14	0.41	1.14:1	12.2	1:17
3	−1	1	−1	1:2	14	1:25
4	1	−0.2	−0.21	2:1	11.6	1:21
5	−1	−1	−1	1:2	10	1:25
6	1	1	−0.25	2:1	14	1:21
7	0.1	−0.95	0.1	1.1:1	10.1	1:19.5
8	0.41	−1	1	1.4:1	10	1:15
9	−0.33	1	−0.11	1.33:2	14	1:20.5
10	−0.95	0.1	0.11	1.05:2	12.2	1:19.5
11	−0.95	0.1	0.11	1.05:2	12.2	1:19.5
12	0.10	−0.95	0.1	1.1:1	10.1	1:19.4
13	−0.25	1	1	1.25:2	14	1:15
14	0.1	0.1	−0.97	1.1:1	12.2	1:24.5
15	0.1	0.1	−0.97	1.1:1	12.2	1:24.5
16	1	1	1	2:1	14	1:15
17	1	−0.25	1	2:1	11.5	1:15
18	−1	−1	1	1:2	10	1:15

PO = peppermint oil, MCT = Medium chain triglyceride; HLB = Hydrophilic lipophilic balance; MX = Meloxicam.

**Table 2:** Constraints and goals selected for the optimization of MX-PO-SNEDDS formulation.

Factor	Name	Goal	Lower Limit	Upper Limit
Independent factors	PO: MCT ratio	is in range	−1	1
	HLB value	is in range	−1	1
	MX: Oil mix ratio	is in range	−1	1
Responses	Particle size (nm)	minimize	44	160
	Stability index (%)	maximize	65	95
	Ex vivo Jss (μg/cm^2^ × min)	maximize	1.2	4.1
	Ulcer index	minimize	0.5	2

PO = peppermint oil, MCT = Medium chain triglyceride; HLB = Hydrophilic lipophilic balance; MX = Meloxicam; Jss = steady state flux.

**Table 3:** Solubility data of Meloxicam (MX) in various oils, surfactants, and co-surfactants.

Component	Sample	Solubility of Meloxicam (mg/mL)
Oils	Peppermint oil	440 ± 15
	Oleic acid	330 ± 11
	Soybean oil	310 ± 16
	Sefsol	410 ± 18
	Palm kernel oil	515 ± 20
	Coconut oil	620 ± 29
	Labrafac Lipophile WL 1349	715 ± 37
Surfactants	Tween 80	145 ± 11
	Span 80	204 ± 18
	Labrasol	110 ± 10
	Triton	125 ± 11
	Cremophor	97 ± 8
	Kollifor	60 ± 7
	Polyglyceryl oleate	70 ± 8
	Plurol	80 ± 5
Co-surfactants	PEG 200	110 ± 11
	PEG 400	170 ± 15
	Propylene glycol	90 ± 7
	Transcutol	75 ± 7
	Glycerol	60 ± 8
	Ethanol	130 ± 10

**Table 4:** The independent factors and responses for the design of experiments.

Run	Factor 1	Factor 2	Factor 3	Response 1		Response 2		Response 3		Response 4	
	A:PO: MCT Ratio	B:HLB Value	C:MX: Oil Mix Ratio	Particle Size (nm)		Stability Index (%)		Ex Vivo J_ss_ (μg/cm^2^ × min)		Ulcer Index	
				Observed	Predicted	Observed	Predicted	Observed	Predicted	Observed	Predicted
1	1	−1	−1	160	159.93	65	65.47	1.2	1.14	0.5	0.6141
2	0.14	0.14	0.41	96	93.89	90	92.13	2.6	2.47	1.5	1.90
3	−1	1	−1	71	69.07	67	67.02	3.5	3.54	3.5	3.29
4	1	−0.2	−0.21	120	120.17	69	69.55	1.7	1.83	0.5	0.6623
5	−1	−1	−1	151	151.91	79	79.37	1.3	1.25	3.5	3.38
6	1	1	−0.25	86	86.58	75	74.28	3.1	3.01	0.5	0.6042
7	0.1041	−0.95	0.1	145	142.49	94	93.56	1.4	1.51	2	1.97
8	0.41	−1	1	87	90.23	86	86.74	3	2.95	1.5	1.64
9	−0.33	1	−0.1121	84	85.92	88	89.01	3.1	3.06	2.5	2.46
10	−0.95	0.1	0.11	100	100.57	72	71.14	2.3	2.29	3	3.38
11	−0.95	0.1	0.11	99	100.57	71	71.14	2.3	2.29	3	3.38
12	0.1041	−0.95	0.1	143	142.49	95	93.56	1.5	1.51	2	1.97
13	−0.2537	1	1	49	48.86	89	88.02	4	4.02	2.5	2.47
14	0.1	0.1092	−0.97	109	110.30	93	92.11	2	1.98	2	1.81
15	0.1	0.1092	−0.97	111	110.30	92	92.11	1.9	1.98	2	1.81
16	1	1	1	44	43.48	76	76.49	4.1	4.19	1	0.7371
17	1	−0.25	1	65	64.46	71	69.98	3.4	3.34	1	0.7932
18	−1	−1	1	91	89.79	69	69.31	2.8	2.83	4	3.60

PO = peppermint oil, MCT = Medium chain triglyceride; HLB = Hydrophilic lipophilic balance; MX = Meloxicam; Jss = steady state flux.

**Table 5:** ANOVA data for particle size of MX-PO-SNEDDS formulations prepared in various experimental runs.

Source	Sum of Squares	Degrees of Freedom	Mean Square	F-Value	*p*-Value	-
Model	19,155.20	9	2128.36	459.28	<0.0001	significant
A-PO: MCT ratio	19.14	1	19.14	4.13	0.0766	-
B-HLB value	9742.66	1	9742.66	2102.37	<0.0001	-
C-MX: Oil mix ratio	5329.71	1	5329.71	1150.10	<0.0001	-
AB	1.51	1	1.51	0.3261	0.5836	-
AC	52.55	1	52.55	11.34	0.0098	-
BC	590.10	1	590.10	127.34	<0.0001	-
A^2^	63.31	1	63.31	13.66	0.0061	-
B^2^	111.84	1	111.84	24.13	0.0012	-
C^2^	1681.62	1	1681.62	362.88	<0.0001	-
Residual	37.07	8	4.63	-	-	-
Lack of Fit	32.57	5	6.51	4.34	0.1282	not significant
Pure Error	4.50	3	1.50	-	-	-
Cor Total	19,192.28	17	-	-	-	-

PO = peppermint oil, MCT = Medium chain triglyceride; HLB = Hydrophilic lipophilic balance; MX = Meloxicam.

**Table 6:** ANOVA data for stability index of MX-PO-SNEDDS formulations prepared in various experimental runs.

Source	Sum of Squares	Degrees of Freedom	Mean Square	F-Value	*p*-Value	-
Model	1905.49	9	211.72	125.86	<0.0001	significant
A-PO: MCT ratio	1.63	1	1.63	0.9707	0.3534	-
B-HLB value	5.82	1	5.82	3.46	0.1000	-
C-MX: Oil mix ratio	10.68	1	10.68	6.35	0.0358	-
AB	106.98	1	106.98	63.59	<0.0001	-
AC	47.48	1	47.48	28.23	0.0007	-
BC	6.12	1	6.12	3.64	0.0929	-
A^2^	1831.20	1	1831.20	1088.59	<0.0001	-
B^2^	2.03	1	2.03	1.21	0.3039	-
C^2^	6.90	1	6.90	4.10	0.0773	-
Residual	13.46	8	1.68	-	-	-
Lack of Fit	11.96	5	2.39	4.78	0.1139	not significant
Pure Error	1.50	3	0.5000	-	-	-
Cor Total	1918.94	17	-	-	-	-

PO = peppermint oil, MCT = Medium chain triglyceride; HLB = Hydrophilic lipophilic balance; MX = Meloxicam.

**Table 7:** ANOVA data for ex vivo J_ss_ in various experimental runs.

Source	Sum of Squares	Degrees of Freedom	Mean Square	F-Value	*p*-Value	-
Model	14.27	9	1.59	140.66	<0.0001	significant
A-PO: MCT ratio	0.0019	1	0.0019	0.1693	0.6915	-
B-HLB value	6.85	1	6.85	607.59	<0.0001	-
C-MX: Oil mix ratio	3.87	1	3.87	343.53	<0.0001	-
AB	0.0299	1	0.0299	2.65	0.1422	-
AC	0.0689	1	0.0689	6.12	0.0385	-
BC	0.3777	1	0.3777	33.51	0.0004	-
A^2^	0.0763	1	0.0763	6.77	0.0315	-
B^2^	0.1908	1	0.1908	16.93	0.0034	-
C^2^	1.07	1	1.07	94.96	<0.0001	-
Residual	0.0902	8	0.0113	-	-	-
Lack of Fit	0.0802	5	0.0160	4.81	0.1131	not significant
Pure Error	0.0100	3	0.0033	-	-	-
Cor Total	14.36	17	-	-	-	-

PO = peppermint oil, MCT = Medium chain triglyceride; HLB = Hydrophilic lipophilic balance; MX = Meloxicam.

**Table 8:** ANOVA data for ulcer index in various experimental runs.

Source	Sum of Squares	df	Mean Square	F-Value	*p*-Value	-
Model	19.30	3	6.43	96.67	<0.0001	significant
A-PO: MCT ratio	19.18	1	19.18	288.08	<0.0001	-
B-HLB value	0.0220	1	0.0220	0.3303	0.5746	-
C-MX: Oil mix ratio	0.1132	1	0.1132	1.70	0.2132	-
Residual	0.9319	14	0.0666	-	-	-
Lack of Fit	0.9319	11	0.0847	-	-	-
Pure Error	0.0000	3	0.0000	-	-	-
Cor Total	20.24	17	-	-	-	-

PO = peppermint oil, MCT = Medium chain triglyceride; HLB = Hydrophilic lipophilic balance; MX = Meloxicam.

**Table 9:** Optimum formula suggested for the MX-PO-SNEDDS formulation and the predicted and observed responses for the optimized formula.

Independent Factors											
**Factor**			**Name**			**Level**			**Actual Value**		
A			PO: MCT ratio			0.7845			1.78:1		
B			HLB value			1.0000			14		
C			MX: Oil mix ratio			1.0000			1:15		
**Responses**				**Predicted Value**				**Observed Value**			
Particle size (nm)				45.3474				47 ± 3			
Stability index (%)				83.562				85 ± 4			
Ex vivo J_ss_ (μg/cm^2^.min)				4.1284				4 ± 0.6			
Ulcer index (%)				1.03552				1 ± 0.25			

PO = peppermint oil, MCT = Medium chain triglyceride; HLB = Hydrophilic lipophilic balance; MX = Meloxicam; Jss = steady state flux.

**Table 10:** Comparative pharmacokinetic parameters of optimized MX-SNELCs formulation and marketed tablets (mean ± SD, *n* = 6).

Pharmacokinetic Parameters	Optimized MX-SNELCs	Marketed MX Tablets
C_max_ (ng/mL)	1200 ± 150	710 ± 90
T_max_ (min)	30 ± 5.0	90 ± 15
t_1/2_ (h)	16.5 ± 2.5	20.5 ± 3.5
AUC _0-t_ (ng/mL h)	17,557.5 ± 2370.25	11,135.3 ±1632.5
AUC_0-inf_ (ng/mL h)	28,012.2 ± 4311.3	18,312.1 ± 2661.1
K_el_ (h^−1^)	0.042 ± 0.006	0.033 ± 0.004
MRT (h)	23.8 ± 2.5	30.3 ± 2.9
Onset of action	<5 min	30 min
Relative bioavailability (%)	152.97%	-

## Data Availability

Not applicable.
